# Whole-exome Sequencing Helps the Diagnosis and Treatment in Children with Neurodevelopmental Delay Accompanied Unexplained Dyspnea

**DOI:** 10.1038/s41598-018-23503-2

**Published:** 2018-03-26

**Authors:** Wenjia Tong, Yajian Wang, Yun Lu, Tongsheng Ye, Conglei Song, Yuanyuan Xu, Min Li, Jie Ding, Yuanyuan Duan, Le Zhang, Weiyue Gu, Xiaoling Zhao, Xiu-An Yang, Danqun Jin

**Affiliations:** 1 Pediatric Intensive Care Unit, Anhui Provincial Children’s Hospital, Hefei, 230029 P.R. China; 2 Joy Orient Translational Medicine Research Center Co., Ltd, Beijing, 100875 P.R. China; 3 Department of Nephrology, Affiliated Hospital of Hebei University of Engineering, Handan, 056002 P.R. China; 4 Neonatal Intensive Care Unit, Anhui Provincial Children’s Hospital, Hefei, 230029 P.R. China; 5 Department of Neurology, Anhui Provincial Children’s Hospital, Hefei, 230029 P.R. China; 6 Beijing Scientific Operation Biotechnology Co., Ltd., Beijing, 100121 P.R. China; 7 Present Address: Cardiac Center Beijing Chest Hospital, Capital Medical University, Beijing, 101149 P.R. China

## Abstract

Neurodevelopmental delay accompanied unexplained dyspnea is a highly lethal disease in clinic. This study is to investigate the performance characteristics of trio whole exome sequencing (Trio-WES) in a pediatric setting by presenting our patient cohort and displaying the diagnostic yield. A total of 31 pediatric patients showing neurodevelopmental delay accompanied unexplained dyspnea were admitted to our hospital and referred for molecular genetic testing using Trio-WES. Eight genes namely *MMACHC, G6PC, G6PT, ETFDH, OTC, NDUFAF5, SLC22A5*, and *MAGEL2* were suspected to be responsible for the onset of the clinical symptoms and 6 variants were novel. Standard interpretation according to ACMG guideline showed that the variants were pathogenic. Finally, diagnosis of methylmalonic aciduria and homocystinuria, glycogen storage disease, ornithine transcarbamylase deficiency, glutaric acidemia II, mitochondrial complex 1 deficiency, carnitine deficiency, and Schaaf-Yang syndrome was made in 12 out of the 31 patients. Trio-WES is an effective means for molecular diagnosis of infantile neurodevelopmental delay accompanied unexplained dyspnea. As for molecular etiology identification, when routine potential monogenetic inheritance patterns including *de novo*, autosomal recessive, autosomal dominant, and X-linked recessive inheritance analysis is negative, physicians should take into account imprinted genes.

## Introduction

Neurodevelopmental delay is characterized by a significant delay in two or more of the following domains: gross and fine motor skills, language, social and personal activities, and cognition^1^. Sustained neurodevelopmental delay brings great damage to the patients in learning, behavior, and function of their daily life^2^. Dyspnea is a common reason that patients consulting a physician, especially for doctors in intensive care unit (ICU) and pediatric intensive care unit (PICU)^3^. It is documented that dyspnea accounts for 4% of all consultations to a general practitioner^4^. Dyspnea can be induced by a wide spectrum of causes such as pulmonary or cardiac disorder and psychogenic factors. Additionally, neuromuscular disease is found to be an important pathogeny. Mostly, the etiology is evident in line with clinical characterization, physical examination, and accessory examination. In a small group of patients, however, the reason is unexplained after initial assessment^5^. Notwithstanding neurodevelopmental delay accompanied unexplained dyspnea is not frequently encountered clinically, it is highly lethal^6,7^. As a result, it brings great confusion to the physicians together with uncertainty in the treatment and prognosis.

Whole exome sequencing in family trios (Trio-WES) has been widely adopted by clinical laboratories due to its ability to simultaneously analyzing millions of fragments of DNA with a single test comparing with traditional methods^8^. In this study, 31 pediatric patients presenting neurodevelopmental delay accompanied unexplained dyspnea were hospitalized in our hospital and had taken Trio-WES to search for molecular etiology. Herein, we present the diagnostic yield of the patients so as to validate the contribution of Trio-WES for this disease.

## Results

### Characteristics of enrolled patients

To learn about general condition of the patients, clinical information of the cohort was analyzed. The cases enrolled in this study were aged between 1 month and 10 years, with a mean age of 2.6 years (Tables 1 and S1). Male to female ratio was 18:13. As shown in Table 2, among the 31 patients, primary phenotypes like respiratory failure, apnea, gross motor delay, seizures, muscular hypotonia, abnormality of metabolism/homeostasis, and feeding difficulties were noted. Thirteen percent of the cohort had clinical features of more than two of the aforementioned categories.

**Table 1:** Characteristics of families with children with developmental delay accompanied unexplained dyspnea enrolled for WES diagnostic testing.

**Patient ID/Sex/Age of onset**	**Phenotype**	**Gene**	**Disease (OMIM)**	**Mode of Inheritance**	**Variants**	**Inherited From**	**Reported (PMID)**	**Class**
P_02/M/1.2y	Global developmental delay, Muscular hypotonia, Intellectual disability, Dyspnea, Frontotemporal extracerebral space widened	***MMACHC***	Methylmalonic aciduria and homocystinuria, cblC type (# 277400)	AR	c.394C>T(p.Arg132*) c.625insT(p.Val209Valfs*36)	Both	16311595 19573432	**P**
P_05/F/5m	Developmental delay, Eczema, Respiratory failure requiring assisted ventilation	***MMACHC***	Methylmalonic aciduria and homocystinuria, cblC type (# 277400)	AR	c.609G>A(p.Trp203*,80) c.80 A>G(p.Gln27R)	Both	16311595	**P**
P_06/F/1y	Developmental delay, Fever, Anemia, Hyperammonemia, Intermittent generalized erythematous papular rash, Goiter, Hepatosplenomegaly, dystrophia, Increased serum lactate	***G6PC***	Glycogen storage disease Ia (# 232200)	AR	c.648G>T(p.Leu216Leu) c.260delG(p.Trp87Trpfs*15)	Both	9630072	**P**
P_010/M/4y	Neutropenia, Lactic acidosis, Dyspnea, Hyperlipidemia, Hyperuricemia, Decreased liver function, Hepatomegaly	***SLC37A4***	Glycogen storage disease Ib (# 232220)	AR	c.446G>A(p.Gly149Glu) c.572C>T(p.Pro191Leu)	Both	10874322	**P**
P_11/M/8y	Vomiting, Confusion, Hyperammonemia, Low plasma citrulline, Hypoargininemia, Brain hernia, Central cardiovascular failure, Respiratory difficulties	***OTC***	Ornithine transcarbamylase deficiency (# 311250)	XR	c. 386G>A(p.Arg129His)	Mat	8081398	**P**
P_13/M/2.5y	Hypoglycemia, Ketonuria, Hyperammonemia, Increased serum lactate, Motor delay, Exercise-induced muscle fatigue, Pneumonia, Dyspnea, Decreased liver function, Hepatomegaly	***ETFDH***	Glutaric acidemia II (# 231680)	AR	c.473T>G(p.Val158Gly) c.1601C>(p.Pro534Leu)	Both	Novel 18289905	**P**
P_16/M/8m	Global developmental delay, Hyponatremia, Pneumonia, Dehydration, Vomiting, Seizures, Dysphagia, Feeding difficulties in infancy, Cow milk allergy, Abdominal distention, Muscular hypotonia, Hypoplasia of the corpus callosum, Focal T2 hyperintense brainstem lesion, Respiratory failure requiring assisted ventilation	***NDUFAF5***	Mitochondrial complex 1 deficiency (# 252010)	AR	c.836T>G(p.Met279Arg) c.145C>G(p.Arg49Gly)	Both	Novel	**P**
P_18/M/4y	Fever, Vomiting, Coma, Generalized tonic-clonic seizures, Muscular hypotonia, Dyspnea, Cerebral edema, Thrombocytosis	***OTC***	Ornithine transcarbamylase deficiency (# 311250)	XR	c.829C>T(p.Arg277Trp)	Mat	2037279	**P**
P_24/F/1y	Developmental delay, Multiple joint contractures, Muscular hypotonia, Speech articulation difficulties, Feeding difficulties in infancy, Hypoglycemia, Dyspnea	***MAGEL2***	Schaaf-Yang syndrome (# 615547)	AD	c.1628delC(p.Pro543Leufs*159)	Pat	Novel	**P**
P_27/F/10y	Fever, Cough, Palpitations, Decreased Achilles reflex, Pneumonia, Myocarditis, Muscular hypotonia, Hypothyroidism, Decreased liver function, Moderate obstructive ventilatory dysfunction	***ETFDH***	Glutaric acidemia II (# 231680)	AR	c.812A>G(p.Tyr271Cys) c.953T>C(p.Leu318Pro)	Both	Novel	**P**
P_29/F/9m	Global developmental delay, Feeding difficulties in infancy, Pneumonia, Respiratory failure, Muscular hypotonia, Abnormality of carnitine metabolism	***SLC22A5***	Carnitine deficiency, systemic primary (# 212140)	AR	c.1400C>(p.Ser467Cys) c.680G>A(p.Arg227His)	Both	10545605 20574985	**P**
P_31/F/1m	Developmental delay, Multiple joint contractures, Muscular hypotonia, Sleep apnea, Feeding difficulties in infancy, Abnormality of the skin, Dysmorphic facial features	***MAGEL2***	Schaaf-Yang syndrome (# 615547)	AD	c.1996insC(p.Gln666Profs*47)	De novo	27195816	**P**

Note: AR, Autosomal recessive; AD, Autosomal dominant; XR, X link recessive; Mat, Maternally inherited; Pat, Paternally inherited; P, Pathnogenic.

**Table 2:** Clinical phenotypes of the cohort.

Affected individuals	Total
Families	31
Affected children	31
Consanguineous	0
PICU enrollments	31
Encephalopathy	6
Global developmental delay	17
Dystonia	1
Intellectual disability	4
Intrauterine growth retardation	3
Morphological abnormality of the central nervous system	9
Muscle weakness	7
Developmental regression	4
Seizures	13
Failure to thrive	9
Muscular hypotonia	13
Skin rash	5
Abnormality of metabolism/homeostasis	9
Easy fatigability	4
Feeding difficulties	8
Abnormality of the liver	7
Abnormality of the kidney	2
Apnea	12
Dyspnea	5
Respiratory failure	14

### Whole exome sequencing (WES) data

In order to identify the molecular etiology, WES was performed using peripheral blood of the family trios. Detailed sequencing data information of the cohort was illustrated in Table S2. Nucleotide variants were detected by using a pipeline optimized for sensitivity to identify variants, yielding 128244 variants per WES. Data with >10× mean coverage accounted for 97.3% of the whole data. The mean average coverage for the cohort was 142×, ranged from 118× to 179×. A mean of 2678 clinically useful candidate variants were obtained after filtering. With the application of internal normal controls, about 400 variants were identified for each case. The remaining 14% were false positives due to unequal allele fractions, sequence homology, poor mapping scores, and insertion and deletion erroneous calls. The variants that were suspected as potential etiology were confirmed by Sanger sequencing. Generally, two to six variants were applied to Sanger sequencing for each family trio and finally, the results showed that more than 90% of variants were positive.

### Genomic diagnostic results

**Figure 1:**
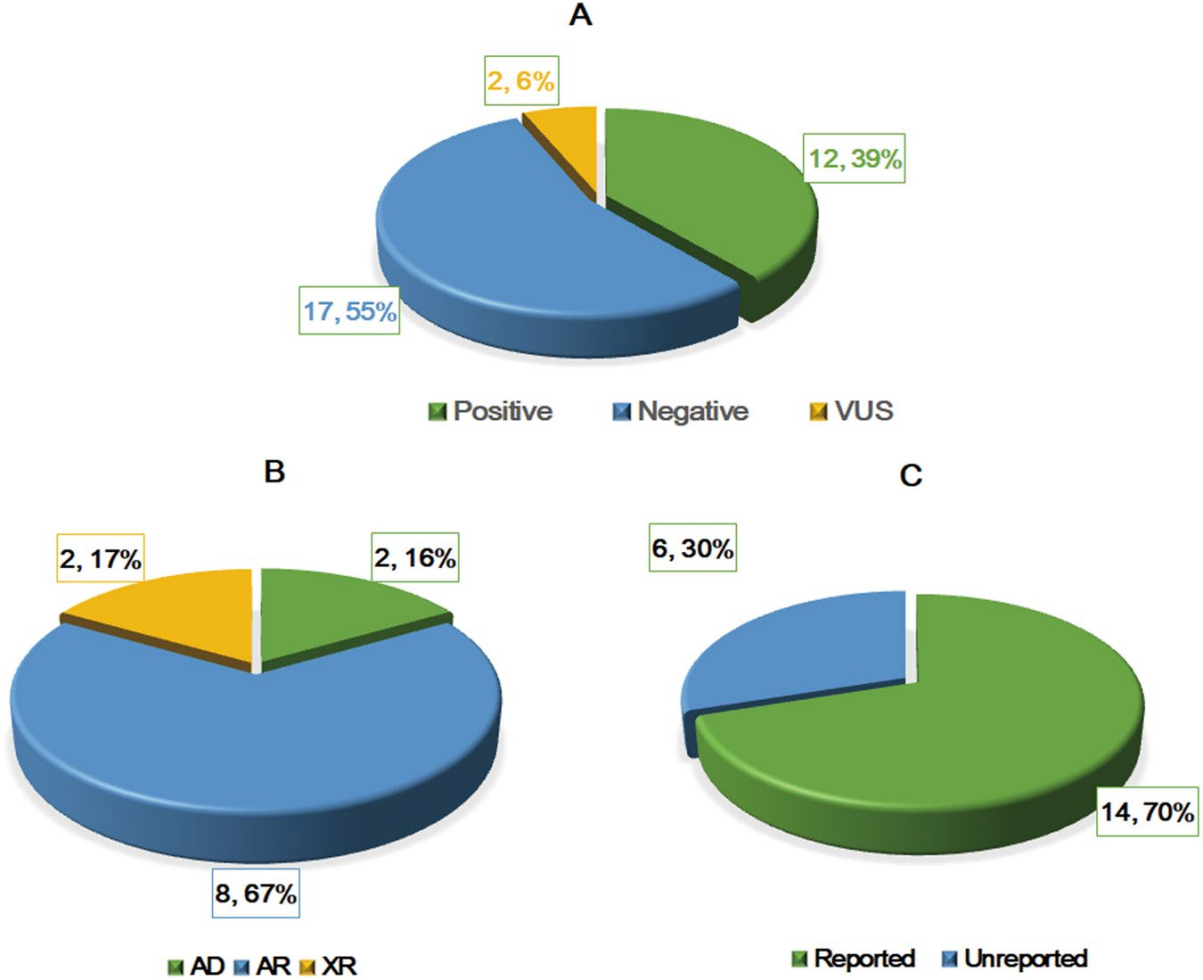
Diagnoses and inheritance patterns in 31 families tested by WES. (**A**) Diagnostic outcomes in 31 patients. (**B**) Inheritance pattern in 12 the families with positive diagnosis. (AR, Autosomal recessive; AD, Autosomal dominant; XR, X link recessive). (**C**) Thirty percent of the pathogenic mutations were previously unreported in the peer-reviewed literature and variant databases.

Diagnoses and inheritance patterns of the 31 families are demonstrated in Fig. 1. Finally, a molecular diagnosis was made in 12 out of the 31 patients (38.7%). Among the 12 patients, 2 cases were diagnosed as methylmalonic academia and homocystinuria (cblC type), 2 cases were identified as glycogen storage disease (Ia and Ib), 2 cases were confirmed glutaric academia II, 2 cases were identified as ornithine aminotransferase deficiency, 1 case were considered as primary carnitine deficiency, and 1 case were identified as mitochondrial complex I deficiency, and 2 cases were Schaaf-Yang syndrome. Detailed information including phenotypes, variants, and mode of inheritance for the 12 patients is illustrated in Table 1. Except the patients who were diagnosed as Schaaf-Yang syndrome, the inheritance models of the patients all were recessive heredity and the pathogenic genes were either compound heterozygotes passed from both of the parents or maternally inherited. The patients diagnosed as Schaaf-Yang syndrome were caused by variants in imprinted gene *MAGEL2*. Most of the identified mutations have been reported, however, 6 variants were novel. Associated phenotypes of these 6 variants are described as follows: three variants of *ETFDH* (c.473T>G(p.Val158Gly); c.812 A>G(p.Tyr271Cys); c.953T>C(p.Leu318Pro) were identified in 2 patients with glutaric acidemia, two variant of *NDUFAF5* (c.836T>G(p.Met279Arg); c.145C>G(p.Arg49Gly) in a proband with mitochondrial complex 1 deficiency, and one variant of *MAGEL2* (c.1628delC, p.Pro543Leufs*159) in a patient with Schaaf-Yang syndrome. The information about clinical features and candidate variants of the family trios of negative and uncertain significance patients were illustrated in Table S1.

### Clinical impact of genomic diagnoses

For the cohort, most patients had taken a long time until definite diagnosis. Our results showed that Trio-WES could avoid unreasonable examination, provide accurate diagnosis, enhance diagnosing efficiency, and guide the treatment. For example, for the patient diagnosed with methylmalonic academia and homocystinuria, WES was applied for classification and treatment response prediction, and the proband recovered finally. As for the two patients of multiple glutaric academia, diet adjustment and long-term application of riboflavin, coenzyme Q10, and carnitine was performed according to WES results. Fortunately, clinical symptoms significantly improved, biochemical indexes recovered to normal range, no metabolic disturbance appeared, and muscle strength and exercise tolerance increased. For the patient with glycogen storage disease type I, diet adjustment together with corn starch, vitamin D, vitamin B1, and calcium supplement was undertaken, and finally, metabolic crisis greatly reduced.

### Case examples

#### P16

**Figure 2:**
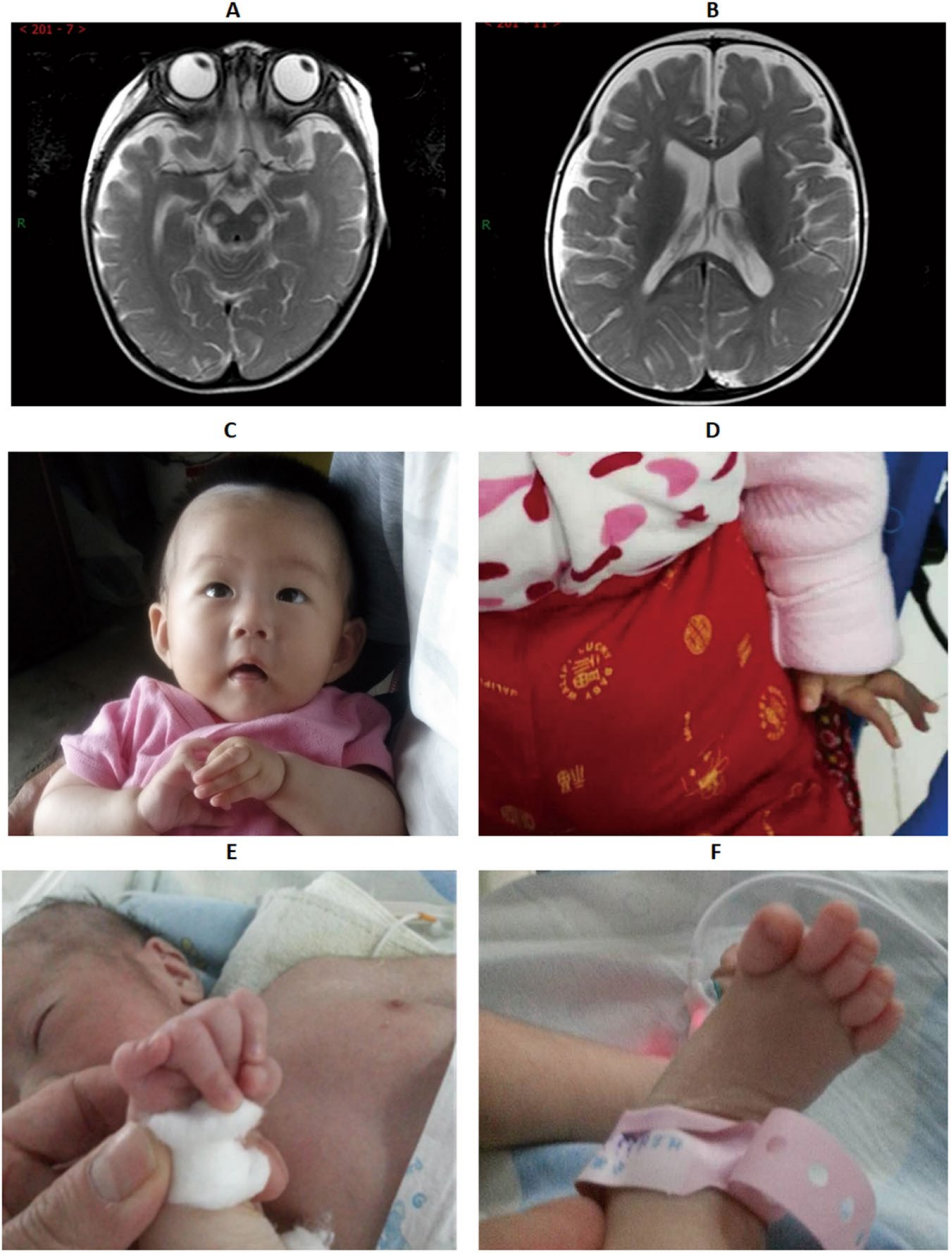
Clinical findings in patients with developmental delay accompanied unexplained dyspnea. For patient 16, long T1 and long T2 signal was revealed by cranial MRI (**A**) and long T1 signal was noted at sagittal medulla oblongata in T1WI. Patient 24 lapsed into unconsciousness, with opening eyes and contracture of fingers (**A**,**B**). Dysmorphic facial features including low set ears and micromandible together with contracture of fingers and toes were noted in patient 31 (**C**,**D**).

P16 was an eight-month old girl when she was admitted to our hospital in April, 2016 due to “cough for 7 days and vomiting for 1 day”. She was the first and only child of healthy non-consanguineous Chinese parents after full term gestation, with a birth weight of 3.2 kg, length of 49 cm, and head circumference of 32 cm. Seizure attacked once after admission and cough and weakened swallowing function were noted. Laboratory examinations showed low sodium and slightly increased lactic acid, pyruvic acid, and blood ammonia. Cranial MRI demonstrated long T1 and long T2 signals with restricted diffusion at the region of bilateral cerebral peduncle and splenium of corpus callosum (Fig. 2A). Additionally, long T1 signal intensity was revealed at sagittal medulla oblongata on T1WI and brain extracellular space of frontal and temporal parts were widened (Fig. 2B). After treated with oxygen inhalation, anti-infection, low sodium correction, and nasal feeding for 12 days, the condition improved and the proband discharged with nasogastric feeding. Unfortunately, she admitted to our hospital once again in May, 2017 because of “lethargy for 1 week”. Glasgow coma scale/score (GCS) was 6 (Best eye response +1, Best verbal response 4+, and Best motor response +1). Breathing irregular rhythm was noted and oxygen saturation could not be maintained, as a result, assisted ventilation was applied. The disease progressed rapidly, and the patient died due to central cardiovascular failure. WES showed compound heterozygosity of c.836T>G(p.Met279Arg) and c.145C>G(p.Arg49Gly) in *NDUFAF5* gene in the proband while the sites were heterozygosity in the parents, arguing that the variants were passed from the parents.

#### P24

Patient24, an 11-month-old girl, was the first and only child of a non-consanguineous Chinese couple. Decreased fetal movement was noted during pregnancy. She was born by cesarean section at full-term, with a birth weight of 3800 g, length of 49 cm, and head circumference of 34 cm. She visited our hospital at 4-month-old due to growth delay and examinations including head MRI, thyroid function assay, limb electromyography, urine gas chromatography-mass spectrometry, blood amino hydroxy acids and carnitine spectra detection, and karyotype analysis found no abnormity. This time, she hospitalized because of fever with vomiting for 1 day and febrile seizures for 4 hours. The child manifested as loss of consciousness, opening eyes, and rhythmic movement of the arms and legs for a few minutes (Fig. 2C). The symptoms could be relieved, however, attacked about every half an hour. She lapsed into mild coma, with the GCS of 9 (eye 2+ verbal 4+ motor 4). Skin mottling was found around all her skin and contracture of fingers was noted (Fig. 2D). Fasting blood glucose test was 1.5 mmol/L (reference value: 3.9–6.2 mmoL/L), indicating hypoglycemia. Immediately after admission, glucose, and midazolam were applied and the seizure stopped quickly. Unfortunately, coarse crackles were noted around the lung and the proband showed dyspnea symptoms and obvious facial cyanosis. She passed away due to cardiovascular failure 5 days after admission.

**Figure 3:**
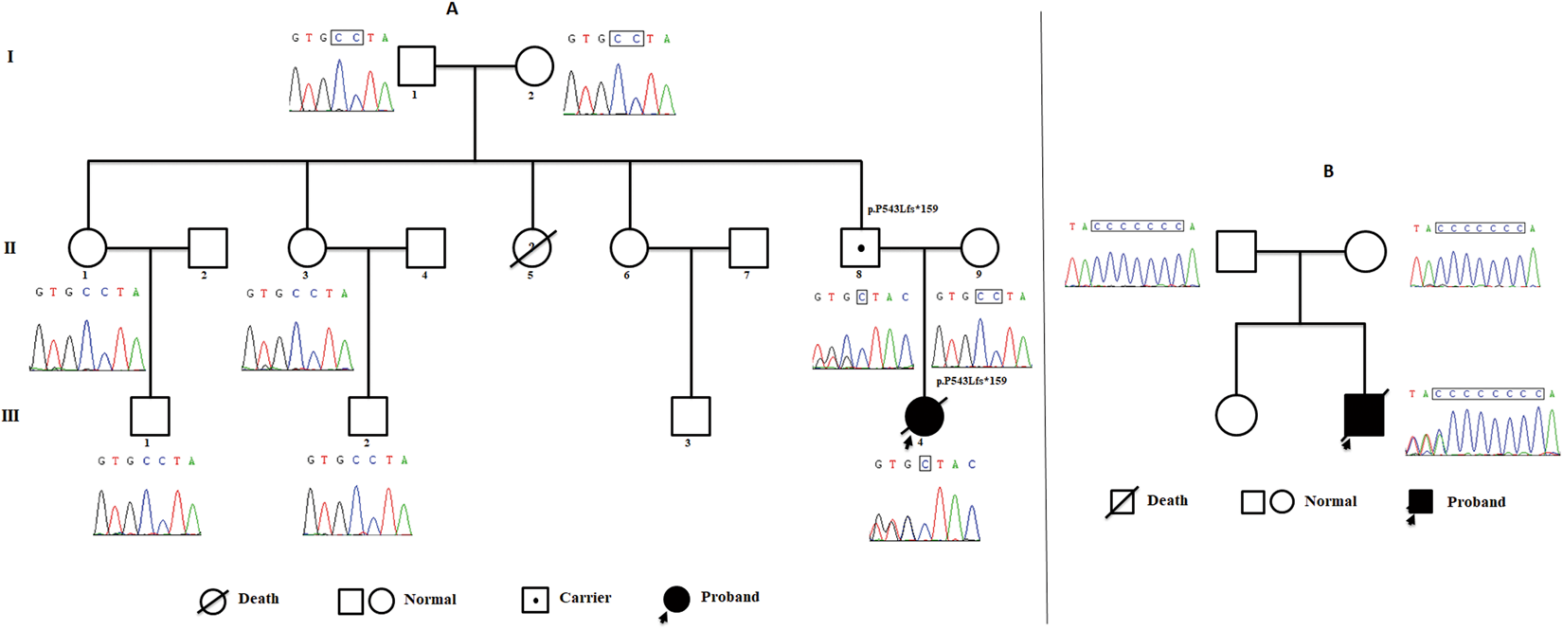
Sanger sequencing of the two pedigrees confirmed *MAGEL2* variants. Patient_24 and her father had the novel heterozygous c.1628delC(p.Pro543Leufs*159) variant in *MAGEL2* while her grandparents, aunts, and cousins were normal (**A**). Patient_31 had a *de novo* c.1996insC(p.Gln666Profs*47) variant in *MAGEL2* (**B**).

The girl was firstly suspected as metabolic disease or mitochondrial disease, but no disease related to mitochondrial gene mutation was found. As a result, some suspicious nuclear genes were screened out (Table S3), however, these genes did not match family segregation analyses or their harmfulness was uncertainty. Additionally, no exons indel was found by checking the NGS data. We recently encountered a patient with genetic disorder caused by imprinting gene^9^, therefore, we focused on the variants of imprinted genes. Finally, we locked into a genetic variant in *MAGEL2*. Trio-WES showed a novel heterozygous c.1628delC(p.Pro543Leufs*159) variant in the patient and her father while the mother showed the “reference allele” at this site. This variant has not been recorded in SNP database (dpSNP, 1000 Genomes, ExAC, ESP, HGMD, Clinvar) or in-house control database. Standard interpretation according to ACMG guideline^10^ showed that the variants were PVS1+PM2+PP4, met the standard of “pathogenic”. Heterozygous mutation in *MAGEL2* was found both in P24 and her father, notably, the girl developed symptoms whereas her father was normal. As *MAGEL2* is reported to be maternally silenced^11^, it is possible to assume that the mutation of the patient was inherited from her grandmother. In order to validate our hypothesis, Sanger sequencing was applied to the pedigree. As illustrated in Fig. 3A, however, the identified site in the grandmother presented reference allele. In addition, her grandfather, aunts, and cousins all were normal in this site.

#### P31

Patient31 was a one month and 14 days old boy when he hospitalized in our hospital. He was the second child born to healthy non-consanguineous Chinese parents after 37 + 4 weeks of gestation. His birth weight was 2820 g, length was 48 cm, and head circumference was 31 cm. Dyspnea appeared without obvious cause, and no fever, cough, expectoration, vomiting, diarrhea, or convulsion was noted. He suffered from poor reaction, sucking rejection, and joint contracture (Fig. 2E,F). Dysmorphic facial features including low set ears and micromandible were found. The scrotum was small, scrotal fold reduced, and the testicle had not fallen. Limb and toe joint contractions were revealed, with extension lag. Limbs muscle tone decreased and the physiological reflex disappeared. Dyspnea was alleviated by tracheal intubation and mechanical ventilation, however, hypotonia still existed. After 1 week of admission, the patient had a stable breathing, and then discharged with a nasal feeding tube. Follow up at two weeks after discharge showed that he was died one week after discharge (two months old).

A *de novo* c.1996insC(p.Gln666Profs*47) variant in *MAGEL2*, which was reported as the mutational hotspot in patients with Schaaf-Yang syndrome^12,13^ was identified by WES. The variant in the family trio was confirmed by Sanger sequencing (Fig. 3B). As *MAGEL2* is an imprinted gene, it is necessary to confirmed that the variant was located on the paternal allele. Unfortunately, the patient passed way and we had no sample to perform further tests. However, combining the clinical manifestation and molecular results, the patient was diagnosed as Schaaf-Yang syndrome.

## Discussion

In this study, we reported high rates of monogenetic disease diagnosis in children showing neurodevelopmental delay accompanied unexplained dyspnea by Trio-WES. We also presented estimates of clinical effectiveness of WES-based diagnoses of this disease. Our results broaded implications for pediatric medicine as neurodevelopmental delay accompanied unexplained dyspnea are a series of highly lethal clinical symptoms. Notably, 2 cases were found with truncating variants in *MAGEL2* gene and were finally diagnosed as Schaaf-Yang syndrome. To the best of our knowledge, this is the first report about patients with Schaaf-Yang syndrome from Asia-Pacific region.

Neurodevelopmental delay is a common symptom found in pediatric clinic that affects about 15% of children between 3 to 17 years old in the United States of America^14^. Next generation sequencing greatly helps the pediatricians in the diagnosis of rare diseases, and the diagnosis rates are from 8% to 45%^12,15,16^. Neurodevelopmental delay is usually associated with other clinical symptoms and the diagnosis rate is generally within this range. For example, in a cohort containing 87 patients behaved as epilepsy and developmental delay, the detecting rate was 19.5%^17^. Our positive rate was 39%, which was consistent with the previously reported results. We consider that the inclusion criteria or the disease might be the major factors that affecting the positive rate.

Many factors such as infection during pregnancy, birth complications, heredity, poison exposure, cancer, abuse, and low socioeconomic status are reported to be associated with increased growth delay^18^. Early identification and intervention for children with retardation could improve developmental outcomes of the sufferers^19^. Accurate diagnosis of pathogens is helpful to treatment, prognosis, medical management, and genetic counseling. With rigorous study, Soden and colleagues argue that comparing with traditional tests, sequencing could decline the economic cost, shortening the diagnosis time, and increase diagnosis effect^12^. In our study, most of the children enrolled in this study came from poor areas of China, and the parents were lack of awareness of health care due to less educated. As a result, reasonable prenatal care during pregnancy and professional health care after birth was lacked. Mild symptoms were neglected and positive treatment was failed during the onset, therefore, it took a long time from the attack to the final diagnosis. For patients who could not make accurate diagnosis clinically, Trio-WES was undertaken. Our results showed relative high diagnosis rates and the therapy upon the diagnosis was very effective.

Case 24 showed metabolic acidosis and increased lactate, therefore, she was initially considered as fatty acid β oxidation disorders or mitochondrial disorders. The proband was finally diagnosed as Schaaf-Yang syndrome by means of Trio-WES. To date, only 30 patients were diagnosed as Schaaf-Yang syndrome worldwide, therefore, the incidence of this disease is very low. However, two patients hospitalized in our department within one year, and hence, we consider that the prevalence of Schaaf-Yang syndrome might be underestimated. Fountain *et al*. demonstrated that joint contracture and autism are the main features of Schaaf-Yang syndrome and *MAGEL2* gene truncating mutation was the molecular pathogeny^20^. In our study, joint contracture was found in the child, however, autism was not yet observed due to her age. Soden and coworkers presented a pair of siblings showing Prader-Willi like syndrome^12^. It is noteworthy that the sisters have hypoglycemia, which was also found in our P24 and the newest report regarding Schaaf-Yang syndrome^21^. This clinical phenotype has not been reported in other cases of the current global known Schaaf-Yang syndrome, hence, it is worth further investigation. Reducing fetal movement and fetal death are reported in patients with Schaaf-Yang syndrome^13,20^, indicating that MAGEL2 might play an essential role during fetal development. As for P24, notwithstanding no obvious abnormal was found by pregnancy test, her mother felt less fetal movement during pregnancy. We consider that as Schaaf-Yang syndrome is a neurodevelopmental disorder, and MAGEL2 plays an important role in the fetal brain, thereby limiting fetal movement^22,23^.

Our study had several limitations. First, the sample size was small, and further study is needed so as to produce huge volume of data for statistical analysis. Second, limited to the level of economic conditions, only patients who had taken Trio-WES were enrolled in this study. Third, part of the negative cases might be false negative and further identification like chromosomal microarray (CMA) or mitochondrial genome should be performed. Especially for cases like P13, P14, P15, P18, and P20, further detection is possible to improve the positive rates.

Collectively, Trio-WES is an effective means for molecular diagnosis of infantile neurodevelopmental delay accompanied unexplained dyspnea. The patients reported here are help to expand the genetic and phenotypic spectrum of the disease. Pediatricians should raise awareness of Schaaf-Yang syndrome and it is possible to pay special attention to imprinted genes when a routine Mendelian analysis failed^24^.

## Materials and Methods

### Study participants

From January 2015 to June 2017, 31 pediatric patients presented neurodevelopmental delay accompanied unexplained dyspnea who were admitted to the PICU at Anhui Provincial Children’s Hospital and had taken trio whole exome sequencing (Trio-WES) were enrolled in this study. They were accompanied by other performances like mental or neurodevelopmental delay, hypotonia, or metabolic crisis (hypoglycemia, lactic acid, metabolic acidosis, and hyperammonemia). The exclusion criteria were as follows: Patients who were less than 35 weeks of gestation age (for patients with too short gestation age, pathological hypotonia might not be reliable); Patients those were with obvious non-hereditary disease (lateral ventricle cysts, hypoxic-ischemic encephalopathy, atelencephalia, intracranial hemorrhage, recurrent intra-spinal canal placeholders, and intracranial infection).

The clinical features of each affected child were ascertained by examination of electronic health records and communication with treating clinicians, translated into HPO terms. This study was approved by the ethics committee of Anhui Provincial Children’s Hospital. All methods were carried out in accordance with the relevant guidelines and regulations. Informed consents were received from all the parents of the participants and informed consent for publication of identifying information/images in an online open-access publication was obtained from the parents.

### Exome sequencing

Peripheral blood from the family trios was subjected to WES and bioinformatics analyses. In brief, genomic DNA from 2 ml peripheral blood was extracted, hybridized, and enriched for WES. Library preparation kits with IDT’s xGen Exome Research Panel (Integrated DNA Technologies, San Diego, USA) and were applied to Illumina Hiseq2500 (Illumina, San Diego, USA). Raw image files were processed using CASAVA v1.82. The sequencing reads were aligned to the human reference genome (hg19) using BWA^25,26^ and PCR duplicates were removed by using Picard 1.27-1 (http://picard.sourceforge.net/). Pipeline incorporating MuTect (single-nucleotide variants, SNVs), genome analysis toolkit (GATK)^27^, and Annovar^28^ was employed for variant calling. FASTQ, bam, and variant call format (VCF) files were then used for sequence analysis.

Minor allele frequency (MAF) was checked using 1000 genomes project database^29^, dbSNP, NHLBI exome sequencing project (ESP, http://evs.gs.washington.edu/EVS/), and ExAC database (http://exac.broadinstitute.org). Restricted analyses to non-synonymous and LoF exonic variants with MAF <0.5% were then performed. Non-synonymous, loss-of-function, indel, duplication, and splice site variants were taken for candidate variants identification. Protein biological function was predicted using Provean^30^, MutationAssessor^31^, and PolyPhen-2^32^.

Variants of ACMG guideline categories benign and likely benign were filtered. For each family, potential monogenetic inheritance patterns including *de novo*, autosomal recessive, autosomal dominant, X-linked recessive inheritance, mitochondrial, and, where possible, imprinted gene variation were analyzed. Full penetrance was assumed for the potentially causal variants and variants that were found in the parents or were recorded in any of the abovementioned databases or in our in-house control exomes were excluded as etiology. Once a variant was considered as the etiology of a recessive disorder, manually inspection for coverage and additional variants of the entire coding domain was undertaken using Integrated Genomics Viewer^33^.

### Sanger sequencing

Sanger sequencing was undertaken to confirm the candidate pathogenic or likely pathogenic variants identified by Trio-WES. Amplification was performed with annealing temperature of 56~60 °C. The PCR products were sequenced with ABI 3730XL (Thermo Fisher Scientific Inc, Waltham, USA) and analyzed by DNASTAR 5.0 software (DNASTAR, Inc, Madison, USA).

## Electronic supplementary material

Supplemental table S1

Supplemental table S2

Supplemental table S3
