# Supplementary material for: Whole-exome Sequencing Helps the Diagnosis and Treatment in Children with Neurodevelopmental Delay Accompanied Unexplained Dyspnea
Source: Sci Rep. 2018 Mar 26;8:5214. doi: 10.1038/s41598-018-23503-2 (PMC5980106; doi:10.1038/s41598-018-23503-2)
Supplement: Supplementary file 2 — Supplemental table S2 [file 41598_2018_23503_MOESM2_ESM.docx]

**Whole-exome Sequencing Helps the Diagnosis and Treatment in Children with Neurodevelopmental Delay Accompanied Unexplained Dyspnea**

Wenjia Tong^1^, Yajian Wang^2^, Yun Lu^3^, Tongsheng Ye^4^, Conglei Song^5^, Yuanyuan Xu^1^, Min Li^1^, Jie Ding^1^, Yuanyuan Duan^1^, Le Zhang^1^, Weiyue Gu^2^, Xiaoling Zhao^1^, Xiu-An Yang^6*^, & Danqun Jin^1*^

^1^Pediatric Intensive Care Unit, Anhui Provincial Children’s Hospital, Hefei 230029, P.R. China; ^2^Joy Orient Translational Medicine Research Center Co., Ltd., Beijing 100875, P.R. China; ^3^Department of Nephrology, Affiliated Hospital of Hebei University of Engineering, Handan 056002, P.R. China; ^4^Neonatal Intensive Care Unit, Anhui Provincial Children’s Hospital, Hefei 230029, P.R. China; ^5^Department of Neurology, Anhui Provincial Children’s Hospital, Hefei 230029, P.R. China; ^6^Beijing Scientific Operation Biotechnology Co., Ltd., Beijing 100121, P.R. China

***Correspondence and requests for materials should be addressed to:** Y. XA. (email: [tkempire@163.com](mailto:tkempire@163.com)) or J. D. (email: jindq69@163.com)

**Supplemental table 2. The detailed sequencing data information of the cohort.**

| **Sample** | **Number of Variants** | | **>10X Coverage (%)** | **Mean depth** |
| --- | --- | --- | --- | --- |
|  | **Prior to filtering** | **After filtering** |  |  |
| 1 | 135145 | 3457 | 98.5 | 143 |
| 2 | 115190 | 2208 | 97.2 | 118 |
| 3 | 102515 | 2276 | 98.3 | 147 |
| 4 | 105487 | 3732 | 99.1 | 145 |
| 5 | 108116 | 3233 | 99.6 | 121 |
| 6 | 143882 | 4632 | 96.3 | 149 |
| 7 | 132668 | 2846 | 97.5 | 153 |
| 8 | 122639 | 2204 | 99.5 | 136 |
| 9 | 133294 | 2156 | 97.3 | 130 |
| 10 | 130775 | 2348 | 96.5 | 125 |
| 11 | 136848 | 2273 | 97.1 | 134 |
| 12 | 115649 | 2112 | 98.2 | 142 |
| 13 | 117457 | 2102 | 94.6 | 179 |
| 14 | 109798 | 2169 | 96.8 | 175 |
| 15 | 140748 | 2097 | 98.4 | 151 |
| 16 | 144373 | 2212 | 99.2 | 121 |
| 17 | 136548 | 2248 | 96.5 | 177 |
| 18 | 106566 | 3152 | 98.7 | 141 |
| 19 | 146786 | 3527 | 96.3 | 142 |
| 20 | 141480 | 4353 | 97.5 | 134 |
| 21 | 146866 | 2383 | 96.1 | 115 |
| 22 | 101581 | 2298 | 96.8 | 162 |
| 23 | 136856 | 2911 | 96.2 | 133 |
| 24 | 145249 | 2210 | 94.6 | 172 |
| 25 | 149823 | 3114 | 96.5 | 134 |
| 26 | 108016 | 2121 | 98.2 | 141 |
| 27 | 148024 | 2399 | 98.1 | 130 |
| 28 | 120858 | 2277 | 97.3 | 146 |
| 29 | 119177 | 3085 | 95.3 | 157 |
| 30 | 156389 | 2440 | 97.4 | 127 |
| 31 | 116782 | 2457 | 98.6 | 149 |
